# Inflammation in Fabry disease: stages, molecular pathways, and therapeutic implications

**DOI:** 10.3389/fcvm.2024.1420067

**Published:** 2024-06-12

**Authors:** Hibba Kurdi, Lucia Lavalle, James C. C. Moon, Derralynn Hughes

**Affiliations:** ^1^ Institute of Cardiovascular Science, University College London, London, United Kingdom; ^2^ Cardiovascular Imaging Department, Barts Heart Centre, London, United Kingdom; ^3^ Lysosomal Storage Disorders Unit, The Royal Free Hospital, London, United Kingdom

**Keywords:** Fabry disease, inflammation, autoinflammatory, innate immunity, adaptive immunity, biomarker, lysosomal storage disorders

## Abstract

Fabry disease, a multisystem X-linked disorder caused by mutations in the alpha-galactosidase gene. This leads to the accumulation of globotriaosylceramide (Gb3) and globotriaosylsphingosine (Lyso-Gb3), culminating in various clinical signs and symptoms that significantly impact quality of life. Although treatments such as enzyme replacement, oral chaperone, and emerging therapies like gene therapy exist; delayed diagnosis often curtails their effectiveness. Our review highlights the importance of delineating the stages of inflammation in Fabry disease to enhance the timing and efficacy of diagnosis and interventions, particularly before the progression to fibrosis, where treatment options are less effective. Inflammation is emerging as an important aspect of the pathogenesis of Fabry disease. This is thought to be predominantly mediated by the innate immune response, with growing evidence pointing towards the potential involvement of adaptive immune mechanisms that remain poorly understood. Highlighted by the fact that Fabry disease shares immune profiles with systemic autoinflammatory diseases, blurring the distinctions between these disorders and highlighting the need for a nuanced understanding of immune dynamics. This insight is crucial for developing targeted therapies and improving the administration of current treatments like enzyme replacement. Moreover, our review discusses the complex interplay between these inflammatory processes and current treatments, such as the challenges posed by anti-drug antibodies. These antibodies can attenuate the effectiveness of therapies, necessitating more refined approaches to mitigate their impact. By advancing our understanding of the molecular changes, inflammatory mediators and causative factors that drive inflammation in Fabry disease, we aim to clarify their role in the disease's progression. This improved understanding will help us see how these processes fit into the current landscape of Fabry disease. Additionally, it will guide the development of more effective diagnostic and therapeutic approaches, ultimately improving patient care.

## Introduction

Fabry Disease (FD) is a rare genetic disorder. It is characterised not just by lysosomal enzyme deficiencies but also by the impact this has on inflammatory pathways. Affecting both males and females, this X-linked disorder arises due to mutations in the galactosidase alpha (GLA) gene (1–3). This results in the accumulation of glycolipids, globotriaosylceramide (Gb3) and globotriaosylsphingosine (lyso-Gb3) within lysosomes in cells throughout the body. This disrupts normal physiological function including the innate immune response (4, 5), such as antigen presentation (6–8), release of inflammatory mediators, and phagocytosis (6, 7, 9). The substrate accumulation is more than a storage anomaly; it triggers a cascade of inflammatory reactions, disrupting typical cellular functions and setting the stage for multiple systemic manifestations.

Inflammation, the body's innate defence response to harmful stimuli, is central to the pathogenesis of FD. Although inherently protective, this immune response, when awry, can change into a chronic, deleterious state. Innate and adaptive immune responses each contribute uniquely to disease progression and their relationship adds complexity to FD.

**Figure 1:**
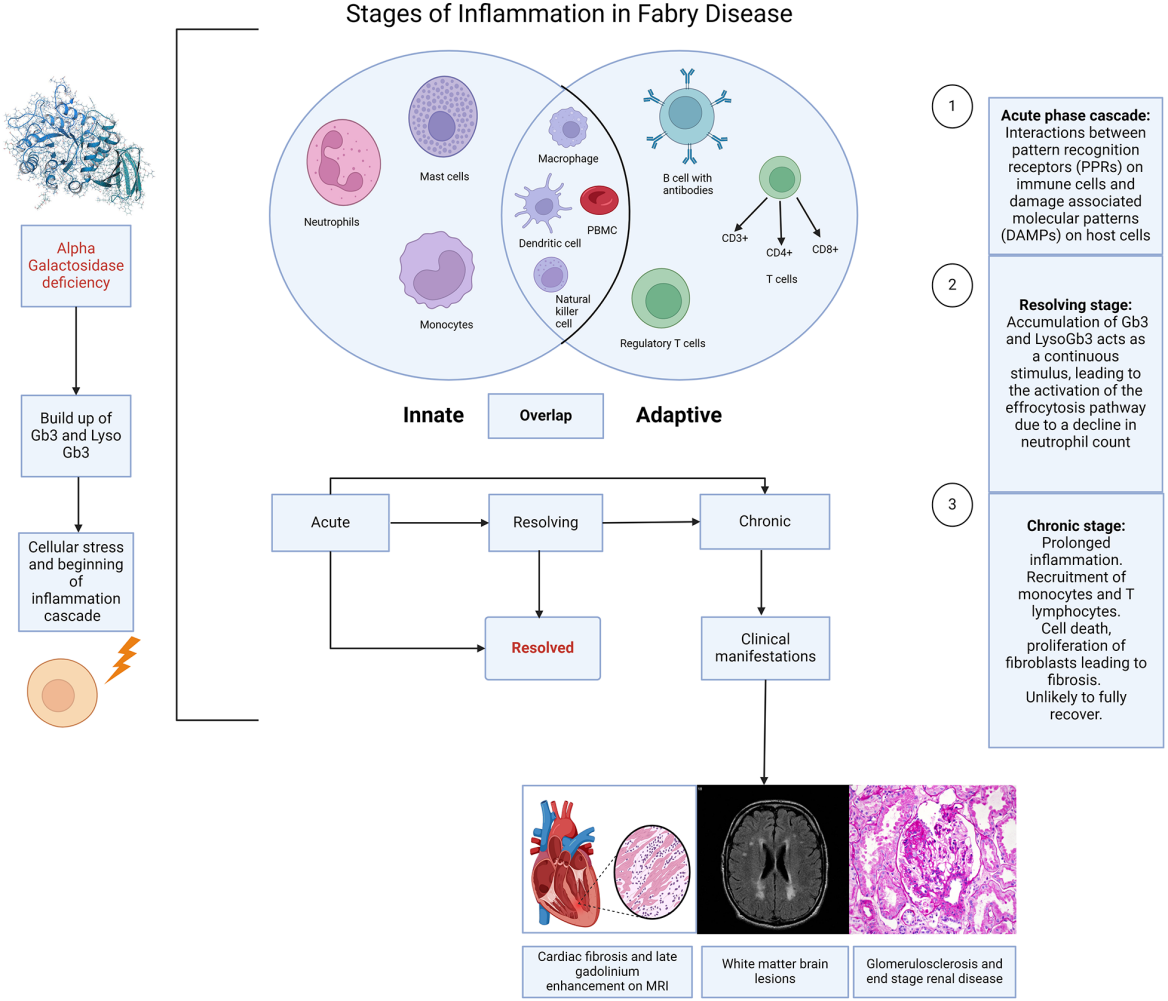
Stages of inflammation in Fabry disease. Caption: illustration depicting the different stages of inflammation in Fabry disease (FD) and the interaction of this with the innate and adaptive immune responses. The immune response in FD is largely initiated by the accumulation of sphingolipids, resulting from the deficiency of alpha galactosidase, which triggers cellular stress and the activation of the inflammation cascade. Acute, chronic and resolving stages are a continuum rather than strictly distinct, although each stage has a recognised hallmark. The innate response involves neutrophils, mast cells, monocytes, macrophages, dendritic cells, and natural killer cells. The adaptive response includes T cells (CD3+, CD4+, CD8+), regulatory T cells, and B cells with antibodies. There are also overlaps between the innate and adaptive immune responses, suggestive of an autoinflammatory component to Fabry disease. (Created with Biorender.com).

**Figure 2:**
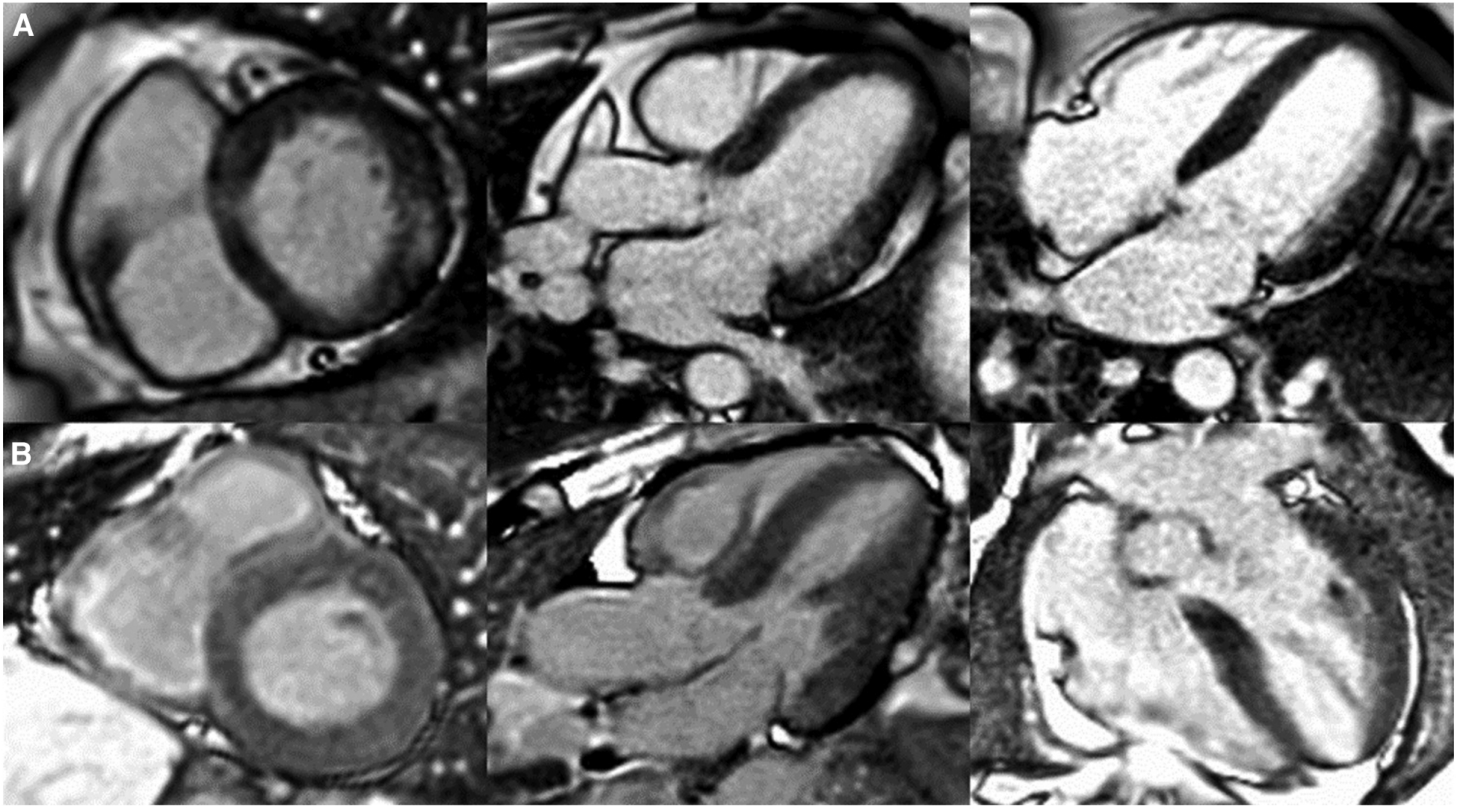
Patterns of cardiac late gadolinium enhancement (LGE) via MRI in Fabry disease. Caption: cardiovascular magnetic resonance LGE images captured using phase sensitive inversion recovery sequences with motion correction. Panel (**A**), left to right: basal short axis, 3 chamber and 4 chamber views depicting pathognomonic basal inferolateral wall fibrosis in a female patient (60 years old) with no left ventricular hypertrophy (maximal wall thickness = 7 mm). Panel (**B**), left to right: basal short axis, 3 chamber and 4 chamber views depicting lack of LGE, therefore fibrosis in a male Fabry patient (51 years old) with left ventricular hypertrophy (maximal wall thickness = 17 mm in the basal antero-septum).

**Figure 3:**
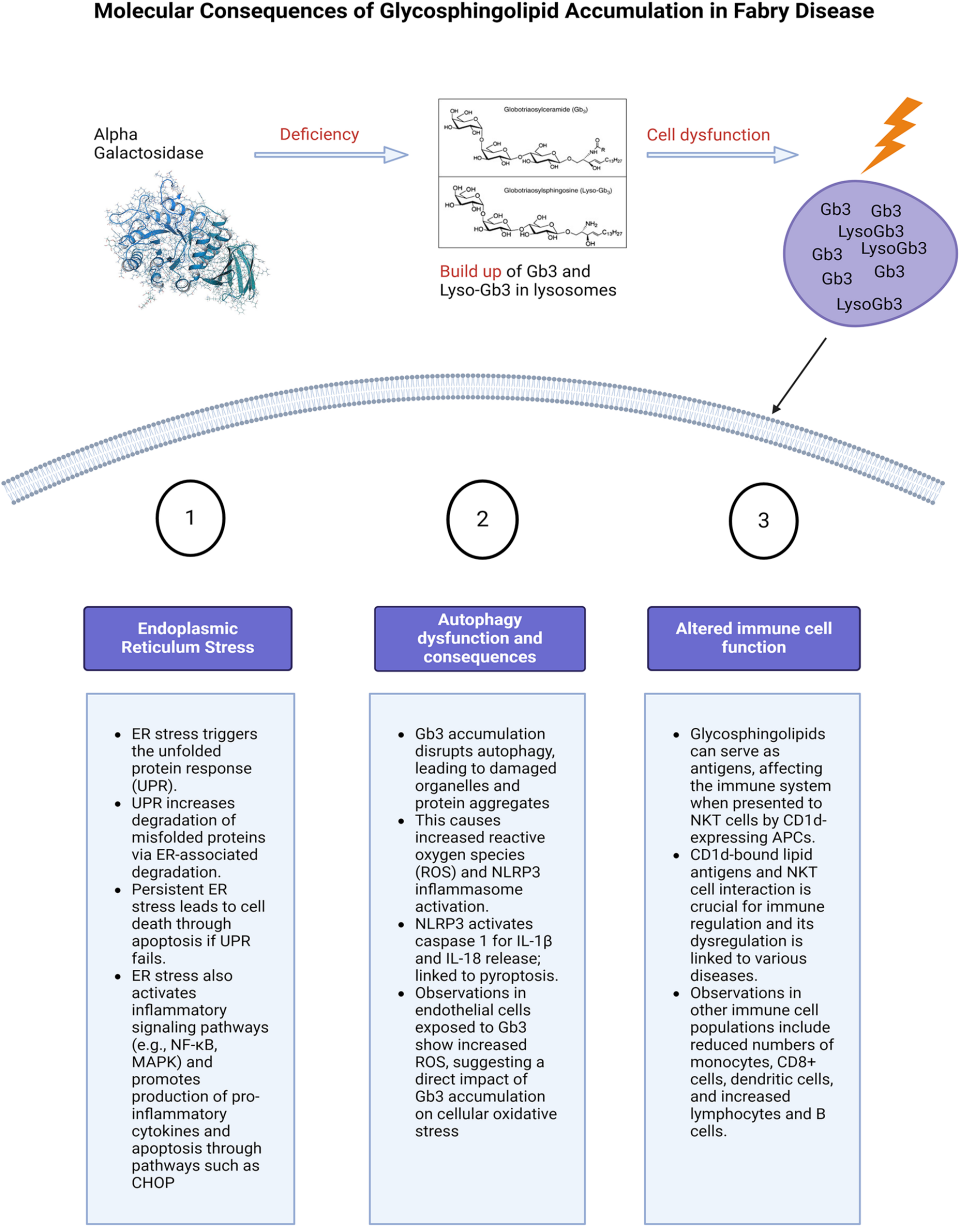
Molecular consequences of glycosphingolipid accumulation in Fabry disease. Caption: figure summarising the cellular stress and molecular consequences of inflammation in FD. This figure illustrates the molecular mechanisms resulting from glycosphingolipid (Gb3 and Lyso-Gb3) accumulation due to alpha-galactosidase deficiency. The main pathways affected are ER stress, autophagy dysfunction, and altered immune cell function. ER stress triggers the unfolded protein response (UPR), leading to protein degradation, cell death, and activation of inflammatory pathways. Autophagy dysfunction caused by Gb3 accumulation results in increased reactive oxygen species (ROS) and the likely activation of the NLRP3 inflammasome, leading to cell death. Additionally, glycosphingolipids act as antigens affecting immune regulation through interactions with NKT cells and altering immune cell populations. Glycosphingolipids can also serve as antigens. ER, endoplasmic reticulum; UPR, unfolded protein response; NF-κB, nuclear factor kappa-light-chain-enhancer of activated B cells; MAPK, mitogen activated protein kinase; CHOP, The C/EBP homologous protein; ROS, reactive oxygen species; NLRP3, NLR family pyrin domain containing 3; Gb3, globotriaosylceramide; APC, antigen presenting cells; NKT, natural killer cells.

The purpose of this review is to chart the course of inflammation as it unfolds in FD. By mapping out the stages, Figure 1, highlighting the consequences of chronic inflammation such as cardiac fibrosis, Figure 2 and delineating the molecular mechanisms at play, Figure 3. By contrasting the roles of adaptive and innate immune responses, we aim to provide a comprehensive overview of inflammatory dynamics in Fabry Disease. Elucidating these mechanisms is pivotal in identifying potential therapeutic interventions, aiming to enhance patient outcomes amidst the complexities of this disorder.

### Innate vs. adaptive immune response in Fabry disease

The demonstration that the accumulation of Gb3 and lyso-Gb3 activates Toll-like receptors (TLRs) (10–12) and inflammasomes provides clear evidence of stimulation of the innate immune response. This provides immediate, non-specific defence, whereas the humoral, also known as the adaptive response is a slower, more targeted mechanism against specific pathogens. Tissue-resident cells including macrophages, neutrophils, and mast cells become activated in response to the cellular stress caused by this accumulation (13, 14). This has been highlighted by previous histological studies from endomyocardial and renal biopsies demonstrating the presence of these cells in FD (15, 16). However, the limited sample size and diversity in disease stages and severity among these studies, coupled with varying treatment backgrounds [including both enzyme replacement therapy (ERT) naïve and non-naïve patients], complicate the direct correlation of these markers with clinical presentations. This variability presents a challenge in accurately determining disease stages, see Figure 1 and understanding the precise role of these markers in FD.

Consequently, chronic inflammation, also described as an autoinflammatory disorder (17), arises from the maladaptation of the innate immune system, which responds to the recognition of damage-associated molecular patterns (DAMPs) in affected cells (14). However, the roles of the innate and adaptive immune responses overlap in chronic inflammation. This is demonstrated not only by the presence of cells such as macrophages which have a role in both immune responses but also due to the role of cells such as dendritic cells and invariant natural killer (iNK) cells that possess characteristics of both NK cells of the innate immune system and T cells of the adaptive immune system and can recognise antigens. These cells have both been implicated in the pathophysiology of inflammation in FD (13, 18, 19). Furthermore Hayashi et al. have demonstrated the ongoing presence of macrophage related markers (CD68, CD163, CD45) on histology in patients with FD and amyloid compared to conditions such as myocarditis (16). Notably, myocarditis presents a continuum of inflammation severity, ranging from mild to severe and can evolve from acute to chronic stages. These findings underscore that the inflammatory profiles observed in Fabry disease and amyloidosis exhibit greater intensity compared to the varied stages and severity seen in myocarditis. This suggests the presence of an intrinsic factor, likely stemming from glycolipid deposits, sustaining inflammation continuously in Fabry Disease and amyloidosis, unlike the fluctuating nature seen in myocarditis (16). The lysosomal accumulation of glycosphingolipids also disrupts regular cellular operations, leading to cellular stress and apoptosis, which mainly stimulates the innate immune system and instigates inflammation pathways like the inflammasome pathway in macrophages (14). However, the myocarditis patients in this study by Hayashi at al may not represent the full spectrum of myocarditis severity.

In the context of Fabry Disease, Gb3 accumulation also leads to the activation of toll like receptors (TLRs) particularly TLR4 (10–12), which in turn stimulates the production of pro-inflammatory cytokines and chemokines, further amplifying the inflammatory response. TLR4 recognises endogenous molecules exposed during cellular injury. Binding of glycolipids such as lyso-GB3 to TLR4 triggers NOTCH1 signalling, which subsequently activates the nuclear factor kappa B (NF-κB) pathway. This results in the production of pro-inflammatory cytokines, giving rise to both systemic and local inflammatory responses (10, 11). In addition to Fabry Disease, TLR4 has been implicated in other lysosomal storage disorders such as mucopolysaccharidoses (20) and Niemann-Pick Type C (21, 22).

The adaptive immune system, mainly composed of T cells and B cells, typically identifies specific pathogens, and fosters long-term immunological memory was thought to play a less dominant role in inflammation in FD. It is traditionally associated with diseases categorised as autoimmune that involve the adaptive immune system mistakenly recognising the body's own molecules as foreign and mounting an immune response against them. However, in FD, self-neutral glycosphingolipids are recognised as antigens by NK cells by CD1d-bearing antigen-presenting cells (23, 24).

This is more typical of an adaptive immune response and there are some studies that demonstrate an association of FD with autoimmune diseases, most commonly latent endocrine disorders related to the thyroid (25, 26). High levels of antibodies associated with autoimmune diseases such as anti-dsDNA and anti-phospholipid have also been reported in FD patients as well as a higher rate of thrombosis hypothesised to be related to the higher levels of anti-phospholipid (27). However, late presenting endocrine dysfunction such as adrenal insufficiency has been seen in a non-immune capacity in FD (28) and may be related to the degree of glycosphingolipid deposition and underlying genetic mutation (29).

Researchers have investigated NKT cells in patients with Fabry disease, finding varying results. While total numbers of CD8+ NKT cells were consistent across studies, the proportion of these cells differed. One study reported a lower proportion of CD8+ NKT cells in Fabry patients compared to healthy controls (30), while another found no significant difference (18). The variations in the results from these two studies could stem from several factors. For instance, the heterogeneity among patients with Fabry disease, in terms of disease severity, specific GLA gene mutations, age, sex, and other individual health considerations, may lead to differing immune responses even in individuals with the same disease. In addition, methodological differences between the two studies could contribute to the discrepancies observed. The techniques employed for sample collection and preparation, sample size and the use of distinct reagents or protocols for identifying and quantifying NKT cells, and the differing statistical analysis methods may all affect the results.

Furthermore, the treatment status of the patients at the time of the study could also impact the number or proportion of NKT cells, thereby influencing the study outcomes. Additionally, some studies observed alterations in other immune cell populations, including reduced numbers of monocytes, CD8+ cells, and dendritic cells, and increased percentages of total lymphocytes and B cells (30). The clinical implications of these immune cell changes in Fabry disease patients remain unclear. Other important considerations arise when taking into consideration the mouse models used in studies investigating immune pathways and treatment efficacy in FD. The α-GAL-A knockout mouse model is often used (31). Although they accumulate GB3 in their organs, there are limitations that mean directly translating this to human models is challenging. This includes a normal lifespan in the mouse models and lack of FD phenotype (31, 32).

While the adaptive immune system's role in Fabry disease inflammation was previously thought to be relatively peripheral, it is worth highlighting that it may have a larger but not fully understood role. For instance, it may participate in responses to cell death and tissue damage. Additionally, it may respond to enzyme replacement therapy, a common treatment for Fabry disease, generating antibodies against the infused enzymes. In addition, cells such as iNKT have roles that bridge both the innate and adaptive immune response.

Complement activation is a recently emerging subject of research in the field of FD and inflammation (33) and is also known to play a dual role in both immune systems. Whilst being a major component of the innate immune response (34), it has a crucial role in augmenting the adaptive immune response through various mechanisms. Key mechanisms include opsonisation, where proteins like C3b mark pathogens for enhanced phagocytosis, thereby facilitating the processing and presentation of antigens by phagocytes (35).

Additionally, the complement degradation product C3d augments B cell efficiency by binding to antigens and enhancing their uptake through complement receptor 2 (CR2), also known as CD21 (34, 36, 37). Anaphylatoxins such as C3a and C5a directly influence lymphocyte activity, promoting cellular responses including proliferation and differentiation (34, 38, 39). Moreover, complement components help recruit and activate more immune cells, such as dendritic cells, which undergo maturation and are crucial for effective antigen presentation and T cell activation (40).

Through these interactions, complement activation also supports the adaptive immune system, highlighting the ongoing continuum between the two. The role of complement activation will be discussed in more detail under the section “Molecular mechanisms of inflammation in Fabry Disease”. Overall, a more thorough understanding of the intricate interplay between the innate and adaptive immune systems in the context of Fabry disease necessitates further research.

### Categorising pathways of inflammation to help define stages of disease

The question “What is inflammation?” is fundamental but challenging to address comprehensively within the confines of a review. As we better understand inflammation, its research, and clinical applications grow clearer. Yet, its definition, especially within lysosomal storage disorders, varies widely. For clarity in disease context, it's useful to categorise inflammation as acute, resolving, and chronic.

#### Acute inflammation

The acute inflammation phase in FD correlates to the activation of the innate immune response (41). What follows is an intricate interaction between surface receptors present on the host tissue cells, called pattern recognition receptors (PPRs) and two subclasses of molecules. These are pathogen-associated molecular patterns (PAMPs), which are associated with various external pathogens but indistinguishable from the host cells, and damage-associated molecular patterns (DAMPs). DAMPs are associated with local host-related tissue injury and damage (14).

The cascade that follows, makes up the acute inflammatory response. In Fabry disease and other disease processes this results from the processes between PPRs and DAMPs. The main cell types in the acute phase are neutrophils and macrophages, followed by lymphocytes in the sub-acute to chronic phases, Figure 1*.* The acute inflammatory stage can also be described as two distinct stages based on cellular response: the vascular component and the cellular component. The result is an increase in vascular permeability and a leucocyte mediated extravasation and phagocytosis.

The sustained activation of these cellular and vascular pathways leads to chronic inflammation, a disease state. Acute inflammation by comparison is short lived, it requires constant stimulation to be sustained as the inflammatory mediators involved are quickly degraded and therefore the process ceases once the stimulus is removed (14). If the stimulus is not removed, there is continuous activation of the acute inflammatory cascade and DAMPs leading to cell death and progression to the chronic stage of inflammation.

#### Chronic inflammation

Chronic inflammation is a slow process that operates in silence, with clinical consequences only apparent at later stages when the damage and resulting clinical sequelae are often irreversible (42). In comparison to acute inflammation, which is time limited, chronic inflammatory responses persist for undefined periods of time, from weeks to years (43). There are also other distinct differences between the two phases. In the acute phase, recruitment of neutrophils from the circulation is the hallmark, whereas the continued recruitment of circulating mono-nuclear leukocytes (including monocytes and populations of T lymphocytes), signal the chronicity of inflammation (43). Accumulation of these cells leads to cell death. Chronic inflammation is also characterised by the proliferation of fibroblasts which by extracellular remodelling and collagen deposition culminating in fibrosis. The emergence of fibrosis can precipitate organ dysfunction, a characteristic of Fabry disease.

Linking inflammation with clinical outcomes presents a challenge. An established hypothesis suggests that accumulated glycolipids in FD instigate a chronic inflammatory response, which subsequently triggers the production of extracellular matrix proteins to aid tissue repair (14). However, if left unchecked, this reparative mechanism can transition into a pathological process, leading to excessive protein deposition and resulting in multi-organ fibrosis. Yet, the direct association between fibrosis and the disease stage remains to be unequivocally demonstrated, confounded by the diverse disease presentations in Fabry disease owing to the varying degrees of residual enzyme activity.

For instance, in females, fibrosis manifests as late gadolinium enhancement (LGE) via cardiac magnetic resonance imaging prior to the development of left ventricular hypertrophy, whereas in males, left ventricular hypertrophy typically precedes fibrosis (44), see Figure 2. LGE is a valuable imaging technique to visualise fibrotic tissues due to their uptake of the gadolinium contrast agent, thereby enabling the identification of the extent and progression of fibrosis. In Fabry disease, LGE serves as an essential tool in disease severity assessment, progression monitoring, and guiding treatment strategies.

Although we will be discussing the inflammatory pathways involved in FD, we will not be discussing these in an organ specific way in the body of this review. However, there are some interesting insights that can be derived from the cardiac manifestations seen in FD.

Cardiac inflammation and fibrosis in Fabry disease are critical factors contributing to the progression of heart dysfunction, with both processes intricately linked to the underlying lysosomal storage disorder and the subsequent immune response. Sphingolipid accumulation leads to activation and infiltration of immune cells (13, 45). This response is influenced by the presence of Gb3 and lyso-Gb3, which serve as antigens, and the activation of the toll-like receptor-4 signalling pathway (13). This leads to local inflammation and promotes pro-fibrotic signalling (13, 14). Additionally, interactions between immune cells and fibroblasts may be critical in promoting the differentiation of myofibroblasts and the subsequent deposition of the extracellular matrix, leading to the cardiac manifestations including hypertrophy and fibrosis (46–48). Endomyocardial biopsy has revealed disorganised and hypertrophied cardiomyocytes, evidence of apoptosis, nitric oxide synthase and accumulation of glycosphingolipids (49). Furthermore, the hypertrophy seen in FD has been linked to ischaemia of the heart tissue (50, 51)^,^ however, more recent advances have highlighted that ischaemia in FD is more likely associated with coronary microvascular dysfunction (52) and some limited evidence that early assessment of coronary flow reserve using cardiac MRI may be an important step in the assessment of the disease; although use of this for prognostication purposes is currently limited due to lack of large scale evidence (53).

The cardiomyopathy seen in FD is more increasingly associated with heart failure with preserved ejection fraction (HFpEF) rather than reduced ejection fraction (HFrEF) (54). In addition, it has been demonstrated that increased levels of pro-inflammatory cytokines such as tumour necrosis factor 1&2, interleukin 6 and matrix metalloprotease (MMP) are seen with increasing severity of cardiac involvement in FD associated with LVH and HFpEF (54, 55) and has differed between FD and healthy volunteers as well as treated FD and non-treated FD. More work is needed on the impact of inflammation on fibroblast activation and proliferation in Fabry disease, the consequences of which are diastolic dysfunction. Inflammation is also thought to be a key player in the cardiac conduction system malfunction seen in FD (56). Although more studies are needed to further elucidate the processes involved, it has been shown that oxidative damage of cardiomyocytes and DNA leads to cell dysfunction and apoptosis which in turn leads to electrical instability and prolonged refractory periods seen clinically on the ECG as conduction disease (49, 57).

Inflammation also plays a part with regards to immune cells of the heart. This is highlighted by endomyocardial biopsy specimens revealing inflammatory macrophage infiltrates (16), T cell interstitial infiltrates noted on CD3 staining (58) and apoptotic myocytes on caspase 3 positive cytoplasmic staining (58). There has also been recent interest into the role of calpains in contributing to hypertrophy and fibrosis diseases of HFpEF. Calpains are cytosolic calcium activated cysteine proteases. It has been hypothesised that they may play a role in hypertrophy through activation of NF-Кβ and fibrosis through activation of growth factor β. Additional research is required to bring these discoveries from the laboratory to clinical practice (59).

In chronic inflammation, distinct families of chemokines regulate the migration of mononuclear cells in comparison to those involved in the acute phase. There are interactions between innate (mononuclear phagocytes) and adaptive (subsets of T lymphocytes) cells. However, overall chronic inflammation morphologically is characterised by the presence of macrophages, as is also evidenced by macrophage infiltrates seen in biopsies of FD patients as described above (16). Macrophages outnumber other cells, although other mononuclear cells such as monocytes are seen. Cytokines released from macrophages regulate the proliferation and activity of fibroblasts. Depending on the pathology involved and cytokine microenvironment, other cells such as CD4+ T helper cells, activated CD8+ helper cells and plasma cells may also be present in cells with chronic inflammation and have been demonstrated in histology of patients with FD.

Insights into the role of macrophages in Fabry disease have been gleaned from studies investigating the renal system. Examination of inflammatory cells in the kidney has shed light on their connection to fibrosis and provided valuable insights into the pathogenesis of this disease process. Following recruitment to the injured kidney, monocytes differentiate into macrophages influenced by the local microenvironment. Macrophages exhibit two main phenotypes: M1 (classically activated) and M2 (alternatively activated) (60, 61). M1 macrophages, induced by Interferon-*γ* and lipopolysaccharide (LPS), produce proinflammatory molecules, exacerbating glomerular injury in conditions like crescentic glomerulonephritis. Conversely, M2 macrophages, induced by IL-4, IL-13, and other factors such as IL-10, transforming growth factor β1 (TGF-β1), and glucocorticoids, promote renal fibrosis through various pathways, including profibrotic growth factor production and activation of fibroblasts into myofibroblasts (61).

The balance between M1 and M2 macrophages plays a crucial role in the progression of renal fibrosis. Additionally, the metabolism of arginine influences macrophage polarisation, with M1 macrophages producing toxic nitric oxide (NO) and M2 macrophages contributing to tissue repair and healing through the arginase pathway (60, 61). Furthermore, recent findings suggest that CD163+ macrophages, commonly associated with the M2 phenotype, may serve as relevant mediators of fibrosis in Fabry nephropathy, potentially inducing TGFβ1 production and apoptotic cell death in tubular cells (61). These insights highlight the importance of understanding the functional roles of macrophage subtypes in renal fibrosis and other FD related end organ dysfunction, such as cardiac (62).

In addition, TGFβ1 and vascular endothelial growth factor (VEGF-A) have also been noted to be biomarkers of FD associated cardiomyopathy, which is the chronic stage of the disease as evidenced by left ventricular hypertrophy, fibrosis and arrhythmias (48). Ivanova et al, identified in a study of 45 FD patients categorised by LVH severity, that firstly Lyso-Gb3 levels correlate with TGF-β1 and VEGF-A (63). In addition, there was no gender-related connection between TGF-β1 and LVH. However, VEGF levels were higher in males with FD and LVH. Females who had electrical abnormalities on the ECG but no obvious LVH also showed increased levels of TGF-β1; thus highlighting this as an effective biomarker of early disease (45, 63). The association of TGF-β1 is seen with cardiac end points such as fibrosis and LVH is seen in other lysosomal storage diseases. For instance, in cardiac biopsies obtained from individuals with MPS type I, an excessive activation of TGF-β signalling was observed, as shown by an increase in the levels of phosphorylated SMAD2/3 (64). These findings were associated with cardiac hypertrophy, stenosis in the coronary arteries, and fibrosis.

It has also been demonstrated that Lyso-Gb3 can result in TGF-β1 secretion through vascular endothelial cell activation. However, using a murine model of adventitial fibroblasts, Choi et al. found that lyso-Gb3 could inhibit proliferation, differentiation, and collagen synthesis (65). This highlights that glycosphingolipid accumulation can result in different outcomes according to cell type (45).

In summary, transformation from acute to chronic inflammation is not a continuum, but rather a paradigm shift. Rozenfeld and colleagues hypothesise that FD can be classed as an autoinflammatory disorder (14). This spectrum of disorders is marked by disruptions in the innate immune system, partially as a result of the recognition of DAMPs within injured cells. Autoinflammatory and autoimmune diseases are distinct. Autoinflammatory conditions do not stem from a breakdown in adaptive immune pathways and do not directly influence lymphocyte function. However, both diseases involve the deactivation of negative regulators and an escalation of inflammatory mediators, including chemokines and adhesion molecules.

#### Resolving inflammation (latent stage)

Resolution of inflammation is now regarded as the third stage of the inflammation pathway. It was initially thought to be a passive process, originating from the depletion of the original injury and therefore the depletion of neutrophils from tissues after the initial infiltration (66). However, distinct biochemical pathways are actively turned-on during inflammation in the resolution phase (67), providing evidence for the role of active biochemical pathways in resolution and possibilities for new therapeutic targets.

Chiang et al. highlight that there are specialised lipids that promote the active phase of resolution, 9 specialised pro-resolving mediators (SPMs) (68) and have come to be known as immunoresolvents or “resolvins”. They promote inflammation resolution by clearance of microbes and promote tissue regeneration via specific cellular and molecular mechanisms. Therefore, these resolvins, their pathways and receptors provide new approaches for treating inflammation associated disease. Pro-resolution pathways promote an adaptive immune response that allows for clearance of infection (68). SPMs are not the only therapeutic targets of interest.

Other pathways of interest include the role of cyclopentenone prostaglandins (69) and those involved in the effrocytosis pathway. Effrocytosis is the pathway by which apoptotic cells are removed by phagocytic cells (70). Driving chronic inflammation down a pro-resolution pathway, inhibiting proinflammatory signals that subvert resolution can be sufficient to trigger spontaneous resolution. This may not apply or be possible in chronic disease where the pathways have been depleted or permanently down regulated. Although these pathways are yet to be studied specifically in the context of Fabry Disease, they are future avenues of consideration, especially as the sub-acute or latent phase of Fabry disease is seen clinically but not well defined.

### Molecular mechanisms of inflammation in Fabry disease

**Figure 4:**
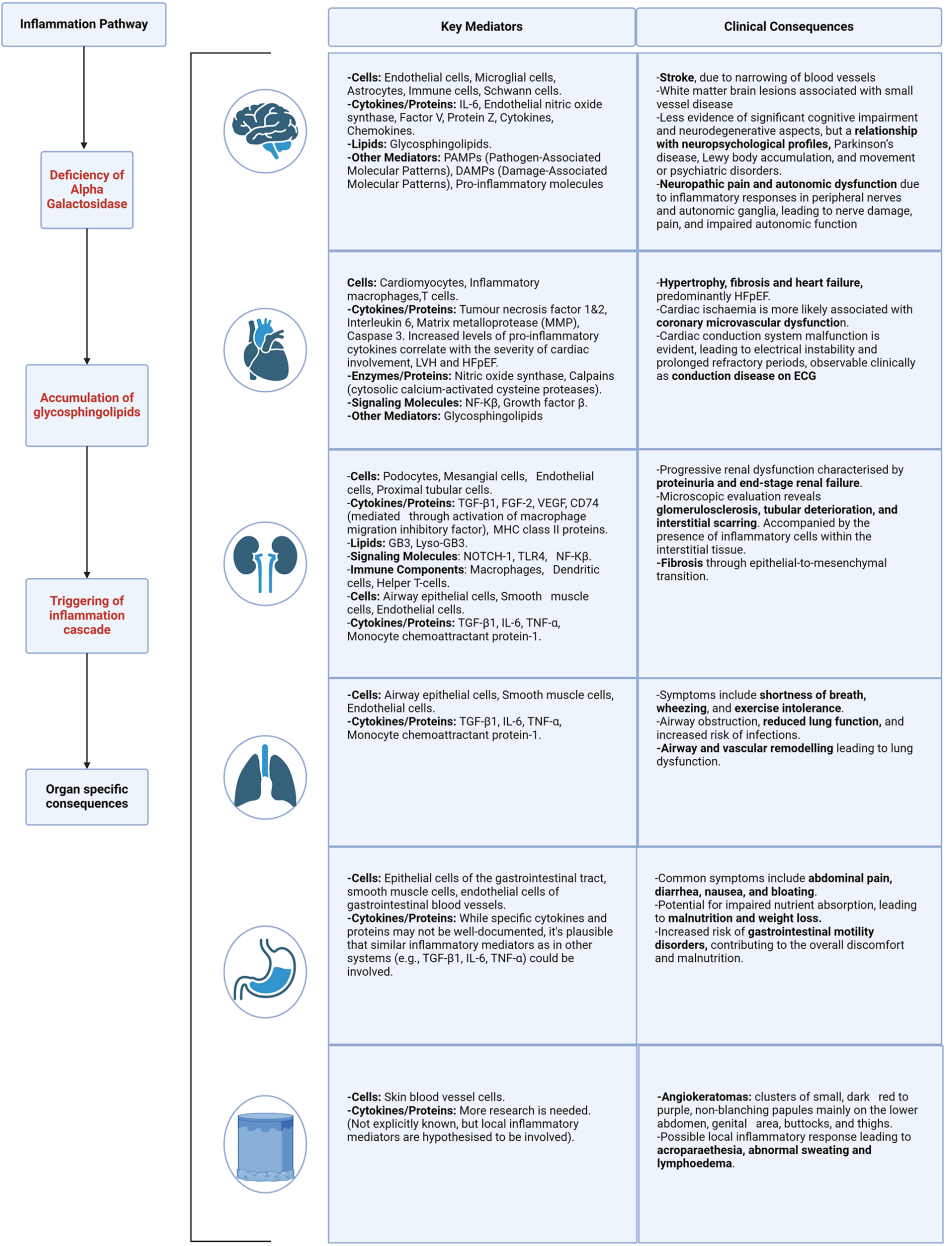
Organ specific mediators and clinical consequences of inflammation. Described for the following body systems—brain, heart, kidney, lung, gastrointestinal and skin. Caption: figure demonstrating the clinical consequences and target organ damage resulting from the inflammation cascade and resulting cellular stress. HFpEF, heart failure with preserved ejection fraction; ECG, electrocardiogram; LVH, left ventricular hypertrophy; NF-kB, nuclear factor kappa B; TGF-β1, transforming growth factor beta-1; fibroblast growth factor 2; VEGF, vascular endothelial growth factor; MHC, major histocompatibility complex; TLR4, toll like receptor 4; TNF-α, tumour necrosis factor alpha.

In the absence of alpha-galactosidase A, Gb3 and lyso-Gb3 accumulate within the lysosomes of many cells and tissues throughout the body. This leads to cellular damage and inflammation responsible for the multi-system symptoms seen in FD (12, 14). In addition to activation of the innate immune system, there are several other mechanisms by which the accumulation of Gb3 and lyso-Gb3 have been reported to trigger inflammation (13). These will be discussed in the following sub-sections and summarised in Figure 3. The organ specific consequences of direct cellular damage are summarised in Figure 4.

#### Endoplasmic reticulum stress and unfolded protein response

Gb3 accumulation triggers endoplasmic reticulum (ER) stress and the unfolded protein response (UPR); enhancing molecular chaperones, reducing protein translation, and increasing misfolded protein degradation via ER-associated degradation (ERAD), Figure 3 (71). Persistent ER stress and UPR failure can lead to apoptosis and activation of inflammatory pathways like NF-κB and MAPK, increasing pro-inflammatory cytokine production (72). Chronic ER stress induces apoptosis, activating signalling pathways such as the C/EBP homologous protein (CHOP) (72). Consolato et al. showed certain α-Gal A mutations, such as missense in Fabry Disease cause ER retention and UPR activation, suggesting a new pathogenic pathway (73). Yet, the reproducibility of results in animal cell-related studies has shown varying results, this will be described in the following paragraphs (73, 74). This phenomenon is also dependent on the underlying structural changes caused by the mutation and the residual activity of the enzyme. Changes in the protein structure can affect how it folds, how stable it is, and how it interacts with other molecules, to different extents depending on the type of mutation.

Furthermore, a study by Nikolaenko et al. demonstrated that lyso-Gb3 both at mild and more severe classical phenotype levels led to increased protein ubiquitination, suggesting that Lyso-Gb3 as well as Gb3 accumulation causes ER stress, a feature of the UPR (74). Proteomic analyses showed that lyso-Gb3 affected cellular systems involved in protein ubiquitination and translation, closely linked to the UPR, such as chaperone/heat shock proteins, cytoskeletal proteins, and synthesis/translation proteins such as heat shock protein 60 and the TRiC complex responsible for the correct folding of newly synthesised proteins and the refolding of misfolded proteins (74). Concluding that lyso-Gb3 exposure disrupts protein translation and folding pathways, leading to ER stress and activation of the UPR.

As described, although persistent ER stress and UPR failure can lead to apoptosis and activation of inflammatory pathways, controversial results occur in different experimental models. For example, different results have been seen in FD peripheral blood mono-nuclear cells (75), compared to α-Gal A missense mutations in HEK293 (Human Embryonic Kidney 293 cell line) knock out mice (73). De Francesco et al. demonstrated that mononuclear cells from untreated Fabry patients presented a higher apoptotic state than patients undergoing ERT. They also demonstrated a high level of caspase 3, however no differences in procaspase3 levels between untreated and treated patients, were seen suggesting that ERT may exert its effect downstream to caspase 3 (75). In addition, variable levels of caspase 4 related to ER stress was found as well as no significant differences between the expression of genes relating to ER stress between untreated patients and normal controls, eliminating the possibility that ER stress is involved in apoptotic cell death in Fabry PBMCs.

#### Impaired autophagy, oxidative stress, and reactive oxygen species

Accumulation of Gb3 in lysosomes interferes with the autophagic process (14, 76), leading to the accumulation of damaged organelles including mitochondria (77) and protein aggregates, triggering inflammation. This leads to increased production of reactive oxygen species (ROS) and activation of the NLRP3 (NOD-, LRR and pyrin domain-containing protein 3) inflammasome. Generation of ROS have been seen in endothelial cells that are exposed to Gb3 (78), and Fabry patients have been noted to have increased oxidative protein damage (79).

The role of the mitochondria in FD pathogenesis and inflammation has been established although the exact mechanisms remain yet to be fully elucidated. Mitochondria play a vital role in cellular energy production, and any disruption to their function can significantly impact conditions like Fabry disease. Lucke et al, were the first to in 2004, report diminished activity in respiratory chain complexes using skin fibroblasts from Fabry patients. This compromised energy production was further corroborated by Ivanova et al. in 2019, who observed variations in total ATP levels in peripheral blood mononuclear cells from individuals with Fabry disease and Gaucher disease (80). This was accompanied by impaired autophagy and accumulation of damaged and ageing mitochondrial cells in peripheral blood mononuclear cells (80). More recently, an *in vitro* investigation by Kim SY revealed that lyso-Gb3 can raise ROS levels via a receptor-interacting protein kinase-3 (RIPK3)-dependent pathway, leading to mitochondrial dysfunction by inhibiting respiratory chain complexes I and III (81). These findings suggest that such mitochondrial alterations are due to substrate buildup.

Energy molecules that directly reflect mitochondrial function such as adenosine triphosphate (ATP) and adenosine diphosphate (ADP), as well as indirect biomarkers of mitochondrial function such as phosphocreatine (PCr) have also been implicated in the pathogenesis of FD and other lysosomal storage disorders (82, 83). Studied have demonstrated reduced levels of phosphocreatine and ATP in FD patients, with levels of phosphocreatine partially restored by ERT (84). This has also been demonstrated through phosphorus 31 magnetic resonance spectroscopy; showing a negative correlation between reduced PCr/ATP ratio and increased LV mass in FD (85). The dysfunction of mitochondrial bioenergetics has also been shown in other lysosomal diseases including Pompe (86) and Gaucher disease (87), suggesting a potential unifying mechanisms that may be a viable therapeutic target.

The integrity of the interaction between lysosomes, autophagy, and mitochondria is essential, and its loss plays a critical role in organ damage in AFD. Schumann et al. (88) observed a dysfunction in autophagy, together with the presence of fragmented mitochondria with altered cristae, in renal cells of patients with FD. In addition, Chou et al. observed a decrease in mitochondrial fatty acid oxidation and a pathological shift towards glycolysis-dominant metabolism for ATP synthesis in iPSCs-derived cells (89).

Chou et al. used Induced pluripotent stem cell-derived cardiomyocytes (iPSC-CMs) obtained from individuals with FD (89). The changes observed were linked to a decrease in the activity of important enzymes responsible for the transportation of fatty acids, specifically mitochondrial carnitine palmitoyltransferase 1 (CPT1) and 2 (CPT2). The alterations were accompanied by an increase in the expression of genes linked to cardiac hypertrophy and a decrease in contractility. Significantly, metabolic disruption continued even after ERT treatment, despite a decrease in Gb3 deposits. This highlights the fact that the development of FD goes beyond just the buildup of sphingolipids and underscores the importance of using additional therapeutic approaches, such as heart failure medication where appropriate alongside ERT. Mitochondria based interventions have been trialled, such as coenzyme Q10 (90) and are discussed in this review by Weissman et al. (45).

Mitochondria also have a large role to play in oxidative stress. Oxidative stress arises when there is an imbalance between the generation of reactive oxygen species (ROS), mainly during mitochondrial oxidative phosphorylation and various enzymatic processes, and the body's ability to detoxify them through its antioxidant system (91). Increased plasma levels of 8-hydroxydeoxyguanosine (8-OHdG), a biomarker for oxidative DNA damage (49, 92) has been found in patients with FD cardiomyopathy. Shen et al. (93) also discovered a lack of tetrahydrobiopterin (BH4), a necessary cofactor for nitric oxide, in heart and kidney biopsies taken from individuals with FD. Other biomarkers of oxidative stress in FD include suppression of superoxide dismutase 2 (SOD2) (94) and glutathione which was found to display sex specific differences in murine models, being downregulated in male mice models more than female (95).

The NLRP3 inflammasome is another emerging area of interest. Given the established link between TLR4 and NLRP3 (96) as well as evidence from Gaucher Disease models (97), highlighting activation of the inflammasome, with subsequent caspase-1 activation, leads to the maturation of IL-1β in Gaucher macrophages and that inflammasome activation in these cells is the result of impaired autophagy. Activation of the NLRP3 is known to result in caspase 1 dependent release of pro-inflammatory cytokines such as IL-1β and IL-18 (98). It has also been linked to gasdermin D mediated cell death termed pyroptosis (99). The discovery of the NLRP3 inflammasome resulted from the uncovering of the gain of function mutation associated with cryopyrin-associated periodic syndrome (CAPS) (100). It has since been implicated in many autoinflammatory diseases that involve chronic inflammation leading to fibrosis and those driven by metabolic dysfunction (101). This includes but is not limited to Alzheimer's disease (102), atherosclerosis (103) and asthma (104). FD appears to have some common inflammation pathways with some of these diseases including cytokine mediated inflammation via IL-1β, mitochondrial dysfunction leading to ROS (105) and lysosomal dysfunction leading to NLRP3 activation (106). FD has been also hypothesised to belong to the “autoinflammatory” class of diseases (14). Targeting the NLRP3 inflammasome and its downstream effects may therefore offer potential therapeutic strategies for managing Fabry disease.

However, in murine models, NLRP3 cytokine blockade still resulted in death (107). Despite the therapeutic targeting of the NLRP3 inflammasome via cytokine blockade, the intervention failed to prevent mortality. This observation underscores the complexity of the NLRP3 pathway and its role in disease pathology. While cytokine blockade effectively reduced IL-1β, it did not address other critical pathways contributing to disease progression, ultimately resulting in death (107). These results highlight the potential limitations of single-target therapies and suggest a need for comprehensive approaches that address multiple aspects of the inflammatory response in these models. It may mean that multiple different approaches to treatment are needed and that these therapies may be best used as adjuvant therapies to existing treatment that replace the defective enzyme, such as ERT or gene therapy.

In addition, Brydges et al. highlight that whilst the major consequence of NLRP3 inflammasome activation is IL-1β mediated, other factors are likely at play (107). In their CAPS murine models, although a T cell response was evident, it was not necessary for displaying the overt disease phenotype and therefore there is likely an innate immunity component to the CAPS syndrome of diseases that may extend to other autoinflammatory diseases, of which FD, as described, is hypothesised to belong to (14).

#### Complement activation

The role of complement activation in the pathogenesis of inflammation in Fabry Disease is an emerging area of research interest. As discussed, it plays a major role in the innate immune system, but also bridges and enhances aspects of the adaptive immune response. A pubmed search using the key words complement and Fabry Disease reveals only 2 relevant studies to date (33, 108). A wider search using lysosomal storage disorders reveals insights into the complement pathway through Gaucher Disease (109–111) and to a lesser extent Niemann-Pick C disease in a murine liver model (112).

The complement system is a vast network of complex cascading protein interactions (34), that are triggered via distinct, yet interconnecting pathways comprised of the classical, alternative and lectin (113). A detailed explanation and analysis of the roles of the complement pathway in health and disease is beyond the scope of this review. However, in 1997, Pandey et al. demonstrated that complement activation in Gaucher Disease (GD), driven by glucosylceramide (GC)-specific IgG autoantibodies formed in the absence of glucocerebrosidase (GCase), leads to C5a production, an anaphylatoxin peptide. This in turn binds to and activates the G protein coupled receptor, C5aR1 (the C5a receptor) which affects the balance of GC synthesis and degradation, promoting further GC storage and triggering immune cell activation and recruitment (109). Thus highlighting the direct role of the complement system in GD and C5Ar1 as a potential therapeutic target. More recently, Laffer et al, have demonstrated the activation of the complement pathway in FD, demonstrated in patients with missense and nonsense mutations, both before and after ERT (33). This study brings to the fore some interesting observations with regards to the relationship between FD genetic mutation type, ERT and complement. A reduction in Lyso-Gb3 compared to baseline was seen with ERT in all groups, suggesting a good treatment response. Patients with nonsense mutations displayed a notable increase in levels of C3a and C5a complement components, suggesting a heightened activation of the complement system. This could be linked to the more severe loss of enzyme function typically associated with these mutations, leading to more intense disease manifestations and immune responses. The relationship between mutation type and response to ERT, is discussed in more detail in the section “enzyme replacement therapy and inflammation”.

Interestingly, in this study by Laffer et al. (33), the presence of anti-drug antibodies (ADAs) is also predominantly observed in patients with nonsense mutations, who also display the elevated levels of C3a and C5a (33). This correlation implies that ADAs may exacerbate immune responses, further stimulating complement activation. The development of ADAs in response to ERT highlights the immune system's reaction to the therapeutic proteins, potentially impacting treatment effectiveness and patient outcomes. Conversely, patients with missense mutations, who generally retain some enzyme function and are less likely to develop ADAs, display variable levels of C3a and C5a in this study (33). This variability suggests a less uniform response to disease and treatment, influenced by the degree of residual enzyme activity and individual immune reactions.

These findings stress the importance of considering genetic and immunological profiles in managing FD. By identifying the type of mutation and ADA status, clinicians can better predict how a patient might respond to ERT and tailor treatment plans accordingly. Additionally, monitoring complement levels could serve as a biomarker for assessing the inflammatory impact of therapy and the overall effectiveness of the treatment regimen.

Dysregulation of the complement system is associated with a myriad of clinical conditions, including haematological—paroxysmal nocturnal haemoglobinuria (114, 115), ocular—age related macular degeneration (116), many renal pathologies—including, but not limited to, C3 glomerulopathy, autoimmune haemolytic uraemic syndrome (HUS), IgA nephropathy, antineutrophil cytoplasmic antibody (ANCA)-associated vasculitis and lupus nephritis (117, 118). As well as the SARS-COV-2 virus (119, 120). This is summarised by Mastellos et al, as well as the current complement derived therapeutic targets and their approval status (34). This includes the approved for clinical use the C5AR1 anatagonist, Avacopan, used in ANCA associated vasculitis (34). Overall, the complement pathway provides new avenues of research and therapeutic targets in lysosomal storage disorders including FD.

#### Exosomes in inflammation

Exosomes are nano-vesicles derived from endosomes; on binding to the plasma membrane, they are excreted into the extracellular space (121, 122). They serve as conduits for the transfer of proteins, lipids, and nucleic acids from donor to recipient cells, thereby modulating their metabolic activities. They are made by many types of immune cells including dendritic cells, T and B lymphocytes, macrophages, mast cells, and reticulocytes, as well as tumour cells and neurons. The exosome cargo is also varied and not only composed of proteins and lipids but also nucleic acids such as miRNA. This includes several inflammatory cytokines including TNF-α (121), which is shown to contribute to inflammation in FD and in particular, myocardial inflammation (54, 55).

Exosomes contain little snapshots of the metabolic material of their origin cell and are present in saliva, blood and urine making them attractive potential biomarkers and targets for therapeutic intervention (121). They are not only mediators of intracellular communication but also have been associated with many inflammatory pathways associated with disease including autoinflammatory conditions such as obesity and Type II diabetes (123, 124), as well as autoimmune disease such as rheumatoid arthritis (125, 126), neurodegenerative disorders such as Alzheimer's disease (127) and cancer (128).

From the observations made from these diseases, advances in the understanding of the role of exosomes in modulating the immune system have made great strides over the last decade. Exosomes that carry cytokines, either within their structure or attached to their surfaces, have the capability to regulate the host immune system. They achieve this by activating B and T cells, enhancing, or inhibiting the production of cytokines in various cells, initiating or suppressing inflammatory responses in specific cells, and encouraging the movement of granulocytes to areas of inflammation.

In this way, it was identified that exosomes could not only act as disease diagnostic and prognostic markers but also as potential avenues for therapeutic approaches. Drawing on these insights, researchers have adapted these concepts to investigate the potential roles of exosomes in Fabry disease, both for enhancing diagnostic accuracy and developing novel therapeutic strategies. Certain microRNAs (miRNAs) in urinary extracellular vesicles (uEVs) showed changes that suggest they play significant roles in the disease's early progression (121). Specifically, miR-21-5p and miR-222-3p were upregulated in patients with stable renal function, while miR-30a-5p, miR-10b-5p, and miR-204-5p were downregulated in those with progressive nephropathy, indicating a connection to key signalling pathways involved in nephropathy development (129). Additionally, miR-126-3p levels were found to increase in plasma EVs of FD patients, with levels rising with age. This miRNA was also linked to premature senescence and increased levels due to glycosphingolipid accumulation in cell studies (130). Another key finding was that exosomal secretion was significantly higher in a FD cell model, and following enzyme replacement therapy with agalsidase-beta, this secretion decreased notably. This change in secretion was interpreted as a cellular response to mitigate the toxic buildup of Gb-3, involving mechanisms like enhanced p53 expression (121, 131).

Although these studies provide interesting insights into the role of exosomes in FD and their potential use as disease biomarkers, there are several limitations that may hinder their translation in to mainstream clinical practice. In the study by Levstek et al, the sample size was very small comprising of 10 FD patients and 10 matched controls. All of these were on disease modifying agents of which the impact on the investigated miRNAs is unknown. Lo Curto et al (130), have a larger cohort of 90 patients but have used treatment naïve patients, highlighting that the generalisability of these study findings across the entire FD cohort is limited due to the wide variations in the population groups. The current treatment avenues exploring exosomes in FD will be discussed under section “Therapeutic targets, future perspectives and challenges”.

##### Enzyme replacement therapy and inflammation

Enzyme replacement and oral pharmacological chaperone therapy are now the cornerstones of treatment in Fabry disease with evidence of organ involvement. Exogenous recombinant enzyme preparations such as agalsidase alpha or beta partially stabilise FD by arresting further accumulation of glycosphingolipids in organs; preventing disease progression and deterioration. It is unclear if ERT also modulates the inflammatory mediators seen in cells and tissues described in this review. A consensus on this is made harder by the different methodologies used in the studies attempting to investigate further. This includes different enzyme preparations, patient populations (sex, age, underling coronary artery disease), treated and untreated FD, stage of disease and non-classical phenotypes that are yet to be fully understood.

Some studies have reported elevated levels of proinflammatory cytokines (79, 132), after ERT treatment and others a reduction (132, 133). However, interpretation of these results is challenging especially due to confounding factors such as concurrent treatment with other anti-inflammatory medication such as statins. New ERT therapies are emerging that provide sustained plasma concentrations, such as Pegunigalsidase (134). However, the nature of the complex interplay between this ERT and the immune system is yet to be determined. As of the time of writing this review there are no studies investigating the use of oral chaperone therapy and its effects on the immune system in FD, apart from indirectly by Braunstein et al. who demonstrated that the UPR could be alleviated by migalastat in transgenic flies expressing mutant α GAL-A variants (135).

Proteomic investigations have evaluated the immunomodulatory impacts of ERT in both animal models and human research. Agalsidase beta was found to normalise the expression of genes related to inflammation, vascular function, and renal function in a study using mouse models (136). Whereas a study conducted on human FD urine proteome demonstrated that agalsidase beta resulted in a decrease in the synthesis of pro-inflammatory proteins, including uromodulin and prostaglandins (137). A further study found that the irregularities in urine proteome indicators of female FD patients were rectified using agalsidase alfa and beta, although the study sample size was small.

De Francesco et al, noted a reduced level of apoptosis in FD PBMCs after agalsidase alfa (75). However, a different study noted no change in lymphocytes, CD19+ cells, CD8+ cells or myeloid dendritic cells between those receiving and not receiving ERT, although these levels were significantly higher in FD patients compared to controls (30). Agalsidase alfa, was shown to normalise upregulated expression of hepatic serum amyloid A1, S100 calcium-binding proteins A8 and A9, and lipocalin 2 in a FD murine model (136). Matafora et al, also showed a reduced level of urinary inflammatory markers such as uromodulin and prostaglandin-H2 isomerase with agalsidase beta treatment (137). A small study by Ko et al. (*n* = 6) demonstrated that agalsidase beta is linked to the upregulation of inflammation-related pathways (innate/adaptive immune pathway, lymphocyte proliferation and leukocyte proliferation were actively regulated under ERT), negative regulation of apoptosis, and activation of the innate immune system, T cell receptor, P38MAPK, IL2RB, and T cell receptor signalling pathways. Conversely, it is associated with the downregulation of the oxidative phosphorylation pathway (138).

Studies have also looked at cardiac parameters of FD, such as the presence of LVH and how this is affected by ERT. In a study by Chen et al, ERT reduced serum levels of IL-6, IL-2, IL-1β, TNF-α, MCP-1, ICAM-1, and sVCAM, and also reduced parameters such as left ventricular mass and indexed left ventricular mass determined by echocardiography (55). This not only emphasises the role of ERT in inflammation but also adds weight to the involvement of these immune complexes in FD associated LVH. All the aforementioned studies highlight that the impact of ERT on inflammatory pathways is varied and complex, also made harder to interpret by the different pathways and immune modulators that are investigated in the different studies.

Although the role of therapy in modulating immune pathways in FD is poorly understood, the recognition of neutralising anti-drug antibodies is an important step in tailoring treatment for maximum efficacy. ERT, which entails infusing a foreign recombinant protein intravenously, can trigger an immune response, leading to the development of specific antibodies against the enzyme (139). Studies show that individuals lacking any endogenous enzyme, termed “cross-reactive immunologic material” (CRIM) negative, are at high risk of such immune reactions following ERT initiation (139–141). Anti-drug antibody formation has been found to be an increased risk in males without any endogenous enzyme (139), higher levels of lyso-GB3 (140, 142, 143) and in some literature the use of agalsidase beta, suggesting there is a link to the increased dosage of agalsidase beta compared to agalsidase alpha (144). The timely identification of ADAs is important as it may lead to a reduction in ERT efficacy and poorer patient outcomes. These findings may also explain why patients with nonsense mutations, and therefore more severe loss of enzyme function had the highest level of the complement anaphylatoxins, C3a and C5a (33).

It is also important to note that this is a separate entity from infusion related reactions (139), which appear to be largely due to IgE independent rather than IgE mediated Type I hypersensitivity reactions (139, 145). Future treatment strategies will focus on further identifying those at risk of anti-drug antibodies by further developing existing laboratory methods to improve sensitivity, including enzyme-linked immunosorbent (ELISA) and electrochemiluminescence (ECL) immunoassays (139). This will allow for more effective screening, confirmation, titration and characterisation of ADAs, thereby enabling the delivery of impactful individualised patient treatment.

### Therapeutic targets, future perspectives, and challenges

The management of FD has significantly evolved in recent years, and emerging therapies like substrate reduction therapy (SRT) and gene therapy promise further transformation. Understanding the links between inflammation and FD pathophysiology is crucial for identifying therapeutic targets and developing novel treatment strategies to mitigate inflammation-associated risks and improve patient outcomes.

SRT aims to reduce glycosphingolipid production by inhibiting their synthesis, potentially alleviating the inflammatory response in FD. Instead of replacing the missing enzyme (as with enzyme replacement therapy), SRT uses small molecules to inhibit the synthesis of glycosphingolipids. This leads to less glycosphingolipids produced, thereby reducing the burden on the cells, and slowing or potentially even stopping the pathological accumulation of these lipids; thereby dampening the inflammation response. One such molecule is Lucerastat (146), an oral glucosylceramide synthase inhibitor, that is under investigation and has shown promising early results. However, in a study on Sandhoff disease, a disorder caused by lysosomal enzyme deficiency, SRT treatment improved survival and protected brain cells in mice without reducing glycosphingolipid buildup. This highlights our limited understanding of glycosphingolipidosis and the urgent need for a symptomatic mouse model for better preclinical testing of Fabry disease therapies.

Gene therapy introduces a functional GLA gene to restore α-Gal A enzyme activity. Gene therapy and ERT both serve to address the root issue of Fabry disease—a deficiency in the α-Gal A enzyme. However, the mechanisms through which they accomplish this differ substantially, and these differences may impact their respective effects on the inflammatory component of Fabry disease.

Enzyme replacement therapy involves the administration of the functional α-Gal A enzyme to a patient. The infused enzyme can reduce the accumulation of glycosphingolipids in the body, hence alleviating symptoms. However, ERT is not a cure, and its benefits are temporary. It needs to be repeatedly administered because the body continually clears the infused enzyme. ERT might not reach all the affected cells effectively due to challenges in crossing biological barriers (e.g., blood-brain barrier, intracellular compartments), which may limit its overall therapeutic efficacy. Recent studies are investigating different administration methods, including subcutaneously, although this far this has only been demonstrated in mouse models (147).

Gene therapy, on the other hand, introduces a functional copy of the GLA gene into the patient's cells. This allows the cells to produce the functional α-Gal A enzyme. Currently most vectors target the liver resulting in expression of alpha galactosidase A from liver cells and secretion into the circulation not dissimilar to exogenously infused ERT. However, novel vectors may allow tissue specific expression. For example, Lentiviral vectors are capable of transducing both dividing and non-dividing cells, offering a durable therapeutic effect. By manipulating the viral envelope proteins, scientists can create lentiviral vectors that target specific cell types (148). Additionally, incorporating tissue-specific promoters into the lentiviral DNA can ensure that the therapeutic gene is only activated in desired tissues. Gene therapy may therefore potentially be expressed by tissues and cells which ERT may not easily penetrate, such as the central nervous system. Furthermore, gene therapy may be more convenient than regular bi-weekly infusions.

While gene therapy has shown promise for treating a range of genetic disorders, it is not without potential risks and challenges. The delivery vectors used in gene therapy, often modified viruses such as adeno-associated viruses (AAVs), have been implicated in cases of immune responses such as haemolytic uraemic syndrome (HUS) (149). When the body detects the viral vector, it may respond as it would to an infection. This can lead to inflammation, as the immune system mobilises to neutralise what it perceives as an invasive pathogen. This inflammatory response can potentially interfere with the effectiveness of the therapy. For example, if the immune system mounts a response strong enough to eliminate the vector, this could limit the duration of the therapeutic effect unless immune modulator therapy is considered. This highlights the importance of careful vector selection. Recent advances in viral vector-based gene therapy have shown promise in preclinical models, with clinical trials underway to assess safety and efficacy in FD patients (77).

Targeting dysregulated cytokines and chemokines, such as TGF-β1, TNF-α, IL-6, and monocyte chemoattractant protein-1, also represent a promising therapeutic avenue. This is a new avenue for research in FD with limited clinical applications currently. However, this approach has been used in other disease states with good clinical success and have now become mainstay of treatment. This includes the use of TNF-α blockers, such as infliximab and adalimumab in Rheumatoid arthritis and Crohn's Disease. Developing inhibitors or modulators of these mediators could alleviate inflammation across various organ systems affected by FD. However, these approaches are not themselves without side effects, which include increased risk of infection due to neutropenia, infusion reactions and anti-drug antibodies (150).

Exosomes have also been explored as potential therapeutic targets in FD, mostly in the role of enzyme delivery. Exosomes are a subpopulation of extracellular vesicles (EVs) (151) which have been investigated as a method for delivering GLA (152). Studies conducted both *in vitro* and *in vivo* demonstrated that these GLA-loaded EVs (EV-GLA) were quickly absorbed and efficiently transported directly to the lysosomes, where they restored enzyme function more effectively than ERT with agalsidase-alfa (152). Moreover, these EVs proved to be well-tolerated and distributed extensively throughout major organs, including the brain, offering a significant benefit over enzyme replacement therapy (ERT), which does not cross the blood brain barrier effectively (153). Additionally, a single dose of EV-GLA administered intravenously successfully reduced Gb-3 levels in key organs such as the kidneys and brain (153).

However, although these new techniques are promising they are currently only in the cell stages of investigation and have yet to be trialled in patients. Whereas the current studies use native exosomes, there is some research ongoing in other diseases using engineered exosomes that may be extrapolated to the FD population in the near future (154). In addition, there are limitations that have been observed from developing EVs for other disease including cancer that are also likely to apply here in addition to the Fabry related heterogeneity of disease.

The isolation and purification of EVs are complex due to their diversity and the presence of other particles in bodily fluids, which is crucial for maintaining purity and avoiding biological effects from contaminants. Additionally, there are no standardised methods for EV production and characterisation, resulting in variability that can impact the effectiveness and safety of these delivery systems.

Efficiently loading EVs with GLA, ensuring their stability during storage and transport, and enhancing their targeting to specific cells affected by Fabry Disease are key technical hurdles. Scalability of production and cost-effectiveness are also major concerns, alongside potential immunogenicity, and safety issues, particularly with EVs derived from allogeneic sources.

Moreover, understanding the *in vivo* behaviour and pharmacokinetics of EVs is limited, complicating their clinical application. Regulatory challenges are significant as well, with guidelines for EV-based therapeutics still under development. Addressing these challenges will be critical for advancing EVs from a novel concept to a practical therapeutic option in clinical settings.

## Conclusion

Fabry disease, characterised by progressive organ damage and increased inflammation due to α-Gal A enzyme deficiency, presents a complex interplay between glycosphingolipid accumulation, inflammation, and organ damage. Despite progress in understanding its pathophysiology, many aspects remain unclear. This review has highlighted the need for comprehensive research and clinical strategies to address inflammation in FD, emphasising the importance of innovative biomarkers, new therapeutic approaches, and integrated care for improving patient outcomes. While current treatments like enzyme replacement and pharmacological chaperone therapy have advanced, the exploration of novel therapies such as substrate reduction, gene therapy, and anti-inflammatory drugs opens new avenues.

However, challenges such as understanding the link between glycosphingolipid accumulation, inflammation, and stage of disease, and developing more effective treatments persist. Fabry disease may possess autoinflammatory characteristics or share autoimmune features that are not yet fully understood. In summary, inflammation plays a critical role in FD, requiring ongoing research to refine treatment and management strategies, with the potential for future breakthroughs in understanding and treating this complex condition.
